# The Gut Microbiota of Pregnant Rats Alleviates Fetal Growth Restriction by Inhibiting the TLR9/MyD88 Pathway

**DOI:** 10.4014/jmb.2304.04020

**Published:** 2023-06-30

**Authors:** Hui Tang, Hanmei Li, Dan Li, Jing Peng, Xian Zhang, Weitao Yang

**Affiliations:** Department of Maternal and Child Health, Changsha Hospital for Maternal & Child Health Care Affiliated to Hunan Normal University, 416 Chengnan Dong Lu, Yuhua District, 410007, Changsha, Hunan, P.R. China

**Keywords:** Fetal growth restriction, TLR9/MyD88, gut microbiota, inflammation, fecal microbiota transplantation

## Abstract

Fetal growth restriction (FGR) is a prevalent obstetric condition. This study aimed to investigate the role of Toll-like receptor 9 (TLR9) in regulating the inflammatory response and gut microbiota structure in FGR. An FGR animal model was established in rats, and ODN1668 and hydroxychloroquine (HCQ) were administered. Changes in gut microbiota structure were assessed using 16S rRNA sequencing, and fecal microbiota transplantation (FMT) was conducted. HTR-8/Svneo cells were treated with ODN1668 and HCQ to evaluate cell growth. Histopathological analysis was performed, and relative factor levels were measured. The results showed that FGR rats exhibited elevated levels of TLR9 and myeloid differentiating primary response gene 88 (MyD88). In vitro experiments demonstrated that TLR9 inhibited trophoblast cell proliferation and invasion. TLR9 upregulated lipopolysaccharide (LPS), LPS-binding protein (LBP), interleukin (IL)-1β and tumor necrosis factor (TNF)-α while downregulating IL-10. TLR9 activated the TARF3-TBK1-IRF3 signaling pathway. In vivo experiments showed HCQ reduced inflammation in FGR rats, and the relative cytokine expression followed a similar trend to that observed in vitro. TLR9 stimulated neutrophil activation. HCQ in FGR rats resulted in changes in the abundance of *Eubacterium_coprostanoligenes*_group at the family level and the abundance of *Eubacterium_coprostanoligenes*_group and *Bacteroides* at the genus level. TLR9 and associated inflammatory factors were correlated with *Bacteroides*, *Prevotella*, *Streptococcus*, and *Prevotellaceae_Ga6A1*_group. FMT from FGR rats interfered with the therapeutic effects of HCQ. In conclusion, our findings suggest that TLR9 regulates the inflammatory response and gut microbiota structure in FGR, providing new insights into the pathogenesis of FGR and suggesting potential therapeutic interventions.

## Introduction

Fetal growth restriction (FGR), also known as intrauterine growth restriction (IUGR), is a complication during pregnancy where newborns are unable to reach their expected size due to genetics-related issues. The high morbidity and mortality associated with FGR pose a significant risk to both the perinatal fetus and the pregnant woman [1, 2]. Although the exact etiology and pathogenesis of FGR are not fully understood, several factors such as impaired placental development and function and disturbances in the maternal gut microbiota have been associated with this condition [3, 4]. Alterations in the human gut microbiota have been linked to the development of various diseases, including obesity, gastrointestinal disorders and autoimmune diseases [5, 6]. In addition, changes in maternal gut microbial species and abundance may also impact maternal micronutrient status and delivery, potentially influencing fetal well-being and pregnancy outcomes [7, 8]. However, the specific molecular mechanisms underlying gut microbiota dysbiosis in FGR patients are yet to be fully elucidated.

Inflammation is a crucial physiological process for maintaining pregnancy and facilitating delivery, but abnormal maternal systemic or local placental inflammation may have detrimental effects on pregnancy outcomes and potentially lead to complications [9, 10]. The proper functioning of trophoblasts, the main cell type of the placenta, is crucial for placental development, and their proliferation, migration and invasion are essential components of this process [11]. However, under abnormal inflammatory conditions, such as elevated levels of pro-inflammatory cytokines and oxidative stress, the placental function can be compromised, leading to the development of FGR [12]. Additionally, clinical studies have observed a higher incidence of IUGR in pregnant women with inflammatory bowel disease (IBD) than those without IBD[13]. Despite these findings, the precise mechanisms via which inflammation induces FGR remain to be clarified.

Toll-like receptors (TLRs) are expressed on the surface of trophoblasts and function as pattern recognition receptors (PRRs) that recognize pathogen-associated molecular patterns (PAMPs) and can elicit rapid inflammation and innate immune response [14]. For instance, TLR4 can be activated by lipopolysaccharide (LPS), leading to pro-inflammatory responses that help protect against pathogen invasion [15]. In this process, LPS-binding protein (LBP) plays a significant role [16]. TLR9, a member of the TLR family, can recognize pathogenic CpG DNA and promote the secretion of pro-inflammatory cytokines in a myeloid differentiating primary response gene 88 (MyD88)-dependent manner following immune system activation [17]. TLRs recruit intracellular TIR domain-containing adapter proteins, such as MyD88, upon ligand binding to trigger innate immune responses. MyD88 is a crucial component of TLR signaling, as its TIR domain can heterodimerize with the TIR domain of TLRs and homodimerize with another MyD88 molecule [18]. TLRs play a vital role in the development and maintenance of placental functions and can influence pregnancy outcome [19]. However, whether TLR9 is involved in regulating the pathogenesis of FGR is currently unknown.

In this study, we performed in vitro and in vivo experiments to investigate the potential involvement of TLR9 in the development of FGR triggered by microbial dysbiosis and inflammation, as well as the underlying mechanisms, to provide valuable insights for identifying alternative strategies for the prevention and treatment of FGR.

## Methods

### Animal Models

Healthy female *Sprague Dawley* rats (9-week-old, weighing 250-275 g) were obtained from Hunan SJA Laboratory Animal Co., Ltd. Rats were housed under standard laboratory conditions (temperature: 22 ± 2°C, humidity: 70%, and a 12-h light/12-h dark cycle). Initially, female and male rats were housed together in a 1:1 ratio, and subsequently, female rats that successfully conceived were included in the following experiments. The observation of a pubic plug or a high concentration of spermatozoa was considered to be day 1 of conception in female rats. The pregnant rats were randomly divided into two groups: the Control group and the FGR group (*n* = 5 per group). Additionally, the pregnant rats were randomly assigned to four groups: the Control group, the FGR group, the ODN1668 group, and the hydroxychloroquine (HCQ) group (*n* = 5 per group). The success rate of the modeling process was approximately 50%. Rats in the Control group were fed regular fodder, while those in the FGR, ODN1668 and HCQ groups were fed low-protein fodder (Wuhan WQJX Biological Technology Co., Ltd., China) [20]. CpG is a ligand for TLR9 [21], CpG ODNs are synthetic oligonucleotides that contain unmethylated CpG motifs in specific sequence contexts, and ODN1668 is a B-class CpG ODN specific for TLR9. Rats in the ODN1668 group were administered 5 mg/kg ODN 1668 via intraperitoneal injection on the 12th day of pregnancy [22, 23]. HCQ is a TLR9 inhibitor that interferes with the interaction between TLR9 and its ligand to inhibit TLR9 expression [24]. Rats in the HCQ group were orally gavaged with 60 mg/kg HCQ (TLR9 inhibitor) on the 12th day of pregnancy, repeated every two days [25]. After 21 days of pregnancy, the rats were euthanized with using an intraperitoneal injection of 150 mg/kg sodium pentobarbital. Maternal serum, cord blood, placenta and feces were collected for subsequent analysis, and the differences in maternal weight, fetal weight and number of fetal rats were recorded. This study was approved by Changsha Maternal and Child Health Hospital Clinical Research Ethics Committee (2022008) and performed in accordance with the guidelines of the animal care and ethics committee.

### Fecal Microbiota Transplantation (FMT)

FMT was conducted as previously described [26]. Briefly, pregnant rats were randomly allocated to one of four groups: the FGR group, the HCQ group, the FMT group, and the HCQ+FMT group, with 5 rats in each group. Fecal samples from FGR rats were collected and resuspended in sterile phosphate-buffered saline (PBS). Rats in the FMT and HCQ+FMT groups received a low protein diet and a weekly administration of 200 μl of the bacterial suspension (~10^8^ bacteria/rat) until the end of the modeling period. FGR rats in the FMT group received the bacterial suspension, while FGR rats in the HCQ+FMT group were given HCQ followed by the suspension.

### Cell Culture

The HTR-8/Svneo cell line, derived from human chorionic cells [27], was obtained from Shanghai Zhongqiao Xinzhou Biotechnology Co., Ltd. The HTR-8/Svneo cells were cultured in Dulbeccós modified eagle medium (DMEM, Gibco, D5796, USA) supplemented with 10% fetal bovine serum (FBS, Gibco, 10099141) and penicillin/streptomycin antibiotics (Beyotime Biotechnology, SV30010, China) and incubated in a 5% CO_2_ incubator (Shanghai SANTN Co., Ltd., DH-160I, China) at 37°C.

To investigate the impact of TLR9 on trophoblast cells, HTR-8/Svneo cells were treated with either the TLR9 activator ODN1668 [28] or the TLR9 inhibitor HCQ. Then, the HTR-8/Svneo cells were exposed to 5 μmol/l ODN1668 or 10 μmol/l HCQ, respectively, while rats in the Control group were given an equivalent dose of media.

### Enzyme-Linked Immunosorbent Assay (ELISA)

The levels of LPS, LBP, interleukin (IL)-1β, tumor necrosis factor (TNF)-α, and IL-10 in the serum of maternal rats and umbilical cord blood (UCB) were measured using ELISA kits (LPS and LBP: ELISA Genie, RTDL00620 and RTEB1783; IL-1β: R&D Systems, RLB00; TNF-α: Proteintech, KE20001, and; IL-10: Proteintech, KE20003) following the manufacturer's instructions. After completion of the reaction, the optical density (OD) values were measured at 450 nm using a microplate reader (HEALES, MB-530, China).

### Quantitative Real-Time PCR (qRT-PCR)

Total RNA was extracted from placental tissue and HTR-8/Svneo cells using TRIzol reagent (Thermo, 15596026, USA). PCR amplification was conducted using the UltraSYBR Mixture (Beijing ComWin Biotech Co., Ltd., CW2601) in a fluorescence quantitative PCR machine (Thermo, Quantstudio1). The PCR program consisted of an initial denaturation step at 95°C for 10 min, followed by 40 cycles of amplification (95°C for 15 sec and 60°C for 30 seconds). *β-actin* was used as an internal reference, and the primer sequences are shown in Table 1.

### Western Blot

Placental tissues and HTR-8/Svneo cells were lysed completely in radio-immunoprecipitation assay (RIPA) buffer (Abiowell, AWB0136, China) and centrifuged (12,000 ×*g*) at 4°C for 15 min to obtain total protein from the supernatant. The protein samples were then separated and transferred onto the nitrocellulose membrane, followed by incubation with their corresponding primary antibodies, including TLR2 (1:1000 dilution, Abcam, ab209217), TLR4 (1:500 dilution, Proteintech, 19811-1-AP), TLR9 (1-5 μg/ml, Abcam, ab134368), MyD88 (1:1000 dilution, Abcam, ab219413), premature IL-1β (1:1000 dilution, Abcam, ab254360), TNF-α (1:5000 dilution, Proteintech, 60291-1-Ig), IL-10 (1:1000 dilution, Abcam, ab33471), PCNA (1:5000 dilution, Proteintech, 10205-2-AP), Ki67 (1:1000 dilution, Abcam, ab16667), MMP9 (1:1000 dilution, Abcam, ab228402), TRAF3 (2 μg/ml, Abcam, ab36988), TBK1 (1: 5000, Abcam, ab40676), IRF3 (1: 1000, Abcam, ab238521), p-IRF3 (1: 5000, Abcam, ab76493), NF-κBp65 (1:1000, Proteintech, 10745-1-AP, USA), p-NF-κBp65 (1:1000, Abcam, ab76302), and β-actin (1:5000 dilution, Proteintech, 66009-1-Ig). Then, they were incubated with HRP-conjugated secondary antibodies, including HRP goat anti-mouse IgG (1:5000 dilution, Proteintech, SA00001-1) or HRP goat anti-rabbit IgG (1:6000 dilution, Proteintech, SA00001-2). The protein bands were visualized using SuperECL Plus (AWB0005, Abiowell) and captured using ChemiScope6100 (CLiNX, China). The relative expression of the target protein was calculated using β-actin as the internal reference.

### 16S rRNA Sequencing

TIANamp Stool DNA Kit (TIANGEN, DP328) was used to extract gut microbial genomic DNA from 200 mg of fecal samples. DNA quality was evaluated using Agilent 4200 TapeStation (Agilent Technologies, USA), subjected to 16S amplicon sequencing by Illumina NovaSeq PE250 to obtain raw data, and the generated raw data underwent separation and filtering processes including de-junctioning, filtering, de-duplication, base correction and removal of chimeric sequences using the Qiime2 software and DADA2 to obtain Feature (collectively referred to as OUT and ASV), as previously reported [29]. The Feature sequences were annotated with species-level taxonomic classification using a naive Bayes classifier and q2-feature-classifier plugin in the silva-138-99 database. Species annotation was performed on each ASV/OUT sequence to generate ASV_table containing corresponding species information and the species-based abundance distribution. Alpha diversity indices and Venn diagram analysis were performed on the ASV_table. Venn diagrams were drawn to determine the specific taxa above the genus level in each group. To assess significant differences in species composition and community structure, analysis of similarity (ANOSIM) was conducted using Bray-Curtis distance analysis and the resulting R-value was obtained. The samples were ranked based on the distance values. Species richness was evaluated using a rank-abundance curve, which visualizes the relationship between relative abundance and species diversity. A histogram displaying the relative richness of species was generated using the R software (version 4.0.2). Differential abundance of taxa at the family and genus level was analyzed using the Wald test. The significance of the microbes that differ between Control and FGR or HCQ was analyzed and represented by *p* values.

### Cell Counting Kit-8 (CCK8) Assay

Cell proliferation was evaluated using a CCK8 kit (Dojindo Laboratories, NU679, Japan). Briefly, the cells were digested with trypsin (Abiowell, AWC0232) and seeded at a density of 1 × 10^4^ cells/well. Subsequently, 100 μl of cell culture medium containing 10% CCK8 was added to each well, and the cells were incubated at 37°C with 5%CO_2_ for 4 h. Following incubation, the OD value was measured using a microplate reader at 450 nm.

### Transwell Assay

To prepare the Matrigel (R&D Systems, 354262), it was diluted with 100 μl of serum-free DMEM medium, and the supernatant was subsequently aspirated. The cells were trypsinized to obtain a single-cell suspension with a concentration of 2 × 10^6^ cells per well. Then, 100 μl of the cell suspension were added to the upper chamber of the transwell system. The lower chamber was filled with 500 μl of complete medium containing 10% FBS. The cells were incubated for 48 h at 37°C. After incubation, the cells were stained with 0.1% crystal violet (Solarbio, G1062) and observed under an inverted microscope (Beijing RXGH Technology Co., Ltd., DSZ2000X).

### Hematoxylin-Eosin (HE) Staining

Placental tissue sections were treated with xylene and different concentrations of ethanol solutions. Hematoxylin solution (AWI0001, Abiowell) was applied for 3 min to stain the tissue. After rinsing, the section was immersed in PBS solution to restore its blue color, followed by staining with eosin solution (AWI0029, Abiowell). The morphological changes and degree of inflammation in the placental tissue were visualized using a microscope (BA210T, Motic).

### Immunohistochemistry (IHC) Staining

To evaluate TLR9 expression in placental tissues, IHC staining was performed. Placental tissue sections deparaffinized using xylene and a series of ethanol gradients (75%-100%). The sections were then immersed in a 0.01 M citrate solution, followed by thermal antigen repair. Endogenous enzyme activity was blocked using 1%periodate. The sections were incubated with a TLR9 antibody (1:200, Abcam, ab37154) overnight at 4°C, then co-incubated with a secondary antibody (1:100, Abiowell, AWS0003) for 30 min. Lastly, the sections were sealed with neutral gel and transferred under a microscope for observation and image acquisition (magnification 100× and 400×). The Image-Pro-Plus software was used to determine positive rates and the TLR9 expression levels.

### Flow Cytometry

Flow cytometry was performed to evaluate neutrophil counts in peripheral blood samples. Initially, erythrocyte lysate (Abiowell, AWC0358b) was added to the blood samples, and the mixture was left undisturbed for 8 minutes to facilitate lysis. The lysate was then subjected to centrifugation, and the resulting cell precipitate was washed with PBS. The cells were resuspended in 100 μl of basal medium and incubated with the CD11b antibody (BioLegend, 201805) for 30 min under light-protected conditions. Following the incubation, 100 μl of the basal medium was added, and the cells were washed with PBS. Finally, the cells were analyzed using flow cytometry (Beckman, A00-1-1102).

### Statistical Analysis

All statistical analysis were conducted using the GraphPad Prism 8.0 software. The data are presented as means± standard deviation. Student’s *t*-test was utilized to compare differences between two groups, while one-way ANOVA was used to analyze the difference among multiple groups, followed by Tukey multiple comparisons test. The correlation between variables was calculated using Pearson's correlation analysis. For 16S sequencing data, non-parametric tests were applied. Differences between groups were analyzed using the Kruskal-Wallis test, while the differences between two groups were assessed using the Wilcoxon test. The threshold *p* < 0.05 was used to determine statistical significance.

## Results

### FGR Induces High Expression of TLR9 and Inflammation Cytokines

**Fig. 1:**
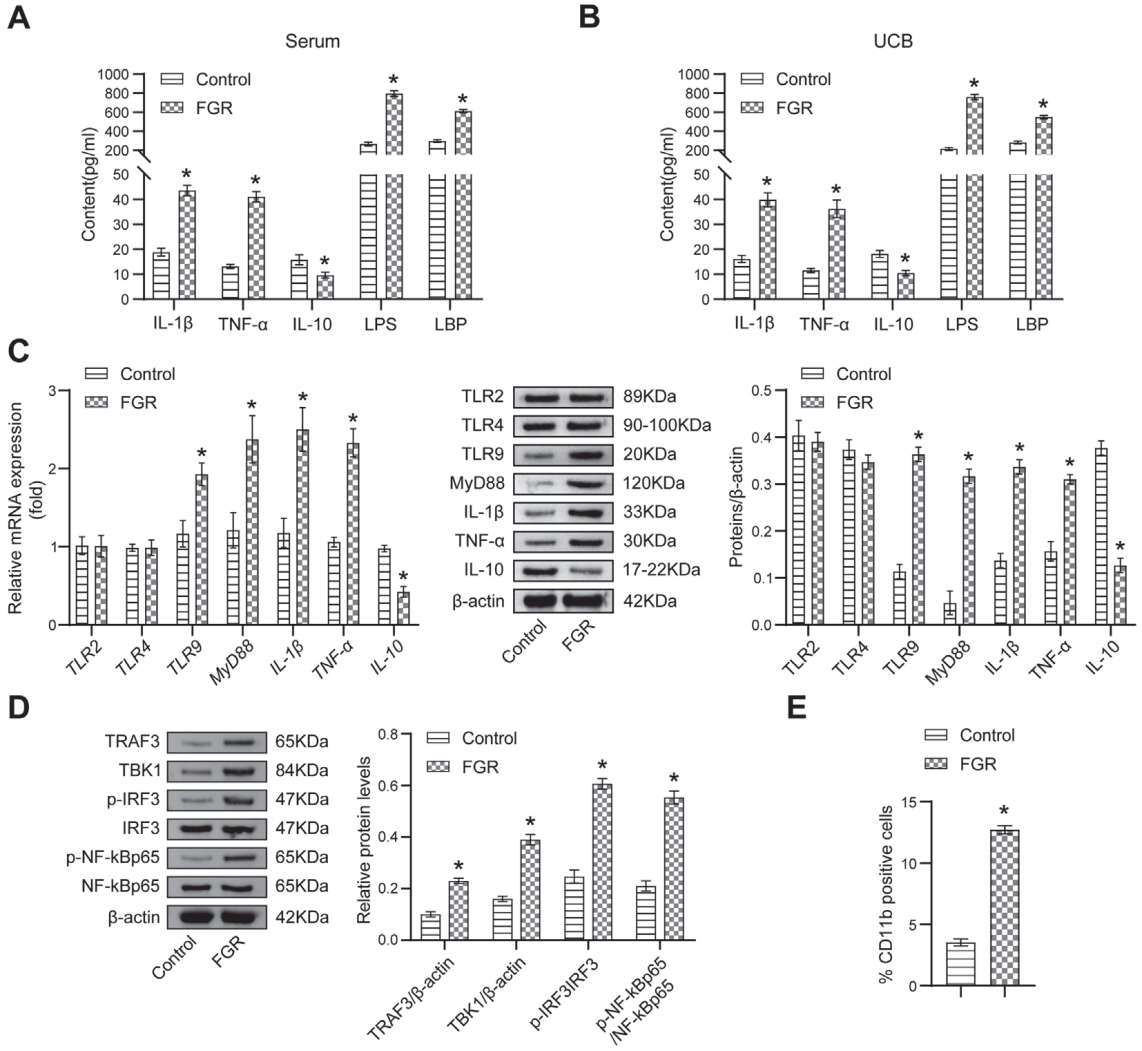
FGR could mediate the expression of inflammatory cytokines and TLR signaling relative regulators. (**A**) ELISA was performed to determine the levels of relative cytokines in maternal rats' serum; (**B**) The expression of relative cytokines in the umbilical cord blood (UCB) was detected by ELISA; (**C**) qRT-PCR and Western blot were utilized to measure the relative mRNA and protein expression of relative cytokines in maternal placenta tissue; (**D**) The protein abundance of TRAF3, TBK1, IRF3 and NF-κBp65 was determined by Western blot; (**E**) The number of CD11b-positive cells was assessed by flow cytometry. *p < 0.05 compared with the Control group.

Since pregnancy complications commonly coincide with abnormal inflammation [9], we investigated the levels of pro-inflammatory cytokines IL-1β and TNF-α, as well as the anti-inflammatory cytokine IL-10, in maternal serum, UCB and placental tissue to examine their association with pregnancy complications. We found that compared to the Control group, the FGR rats exhibited increased levels of premature IL-1β and TNF-α, along with decreased levels of IL-10 (Figs. 1A-1C). Previous studies have suggested the involvement of TLRs in abnormal inflammatory responses [30]. To confirm the role of the TLR signaling pathway in FGR development, we examined the expression levels of relative factors. We observed elevated levels of LPS and LBP in serum and cord blood of the FGR group compared to the Control group (Figs. 1A and 1B). While the expression levels of TLR2 and TLR4 did not differ significantly between the two groups, FGR rats exhibited increased levels of TLR9 and MyD88 (Fig. 1C). Furthermore, we noted elevated levels of TRAF3 and TBK1, as well as increased ratios of p-IRF3/IRF3 and p-NF-kBp65/NF-kBp65 in FGR rats (Fig. 1D). Additionally, the proportion of CD11b-positive cells was higher in the peripheral blood of FGR rats (Figs. 1E and S1A). Taken together, our findings suggest that the activation of the TLR9/MyD88 signaling pathway and subsequent cascade reactions may contribute to the notable differences in inflammatory factors and TLR9 levels observed in FGR rats compared to healthy rats.

### TLR9 Suppresses HTR-8/Svneo Cell Proliferation and Invasion

The placenta is essential for the connection between the mother and fetus, and developmental disorders can disrupt trophoblast cell proliferation, migration, and invasion [31]. We conducted further investigations to explore the impact of TLR9 on trophoblast cell proliferation and invasion. In addition, TLR9 is activated by endogenous mitochondrial DNA (mtDNA) and recognizes GpG DNA [32]. ODN1668 is a B-class CpG ODN that targets TLR9 and acts as an agonist of TLR9. All TLR components have similar dimer structures, and TLRs signaling is dependent on ligand-stimulated dimerization [33]. TLR is an inhibitor of TLR9 and an important molecular target. It disrupts the receptor-ligand relationship, thereby inhibiting TLR9 activation [24]. HTR-8/SVneo cells are a heterogeneous population of trophoblast and mesenchymal cells characterized by rapid cell division, migration and invasion [34, 35], and are commonly used to study the pathological mechanism of major obstetric syndromes involving FGR [36, 37]. Therefore, we utilized ODN1668 and HCQ to treat HTR-8/SVneo cells to investigate the role of TLR9 in trophoblast proliferation and migration.

**Fig. 2:**
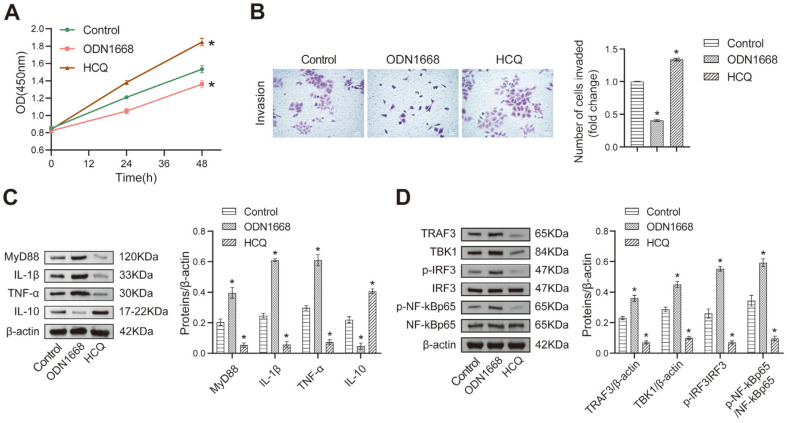
TLR9 could inhibit cell proliferation and invasion and regulate the levels of inflammatory cytokines. HTR-8/Svneo cells were treated with a TLR9 activitor (ODN1668) and a TLR9 inhibitor (HCQ). (**A**) Proliferation ability of HTR-8/Svneo cells after incubation for 0, 24 and 48 h was detected by CCK8 assay; (**B**) Transwell assay was performed to determine the effect of TLR9 on the trophoblast cell invasion; (**C**) The abundance of inflammatory cytokines via Western blot; (**D**) The effects of ODN1668 and HCQ on TRAF3, TBK1, IRF3 and NF-κBp65 expression were determined by Western blot. *p < 0.05 compared with the Control group.

The ODN1668 group exhibited significant decrease in cell proliferation and invasion compared to the Control group, while opposite results were observed in the HCQ group (Figs. 2A and 2B), suggesting that TLR9 could inhibit cell proliferation and invasion, leading to developmental defects in the placenta. Western blot analysis revealed that TLR9 promoted the accumulation of MyD88 in HTR-8/Svneo cells (Fig. 2C). Additionally, in vitro experiments indicated a clear increase in the protein abundance of premature IL-1β and TNF-α in the ODN1668 group, while the levels of IL-10 were decreased and opposite results were observed in the HCQ group (Fig. 2C); thus, indicating that TLR9 could directly mediate the expression of inflammatory factors. Furthermore, ODN1668 increased the levels of TRAF3 and TBK1, as well as the ratio of p-IRF3/IRF3 and p-NF-kBp65/NF-kBp65 compared to the Control group, while HCQ decreased the levels of TRAF3 and TBK1 and the phosphorylation of IRF3 and NF-kBp65 (Fig. 2D). Collectively, these results suggest that TLR9/MyD88 can activate downstream cascade reactions.

### TLR9 Regulates Inflammation Response and Intestinal Permeability

**Fig. 3:**
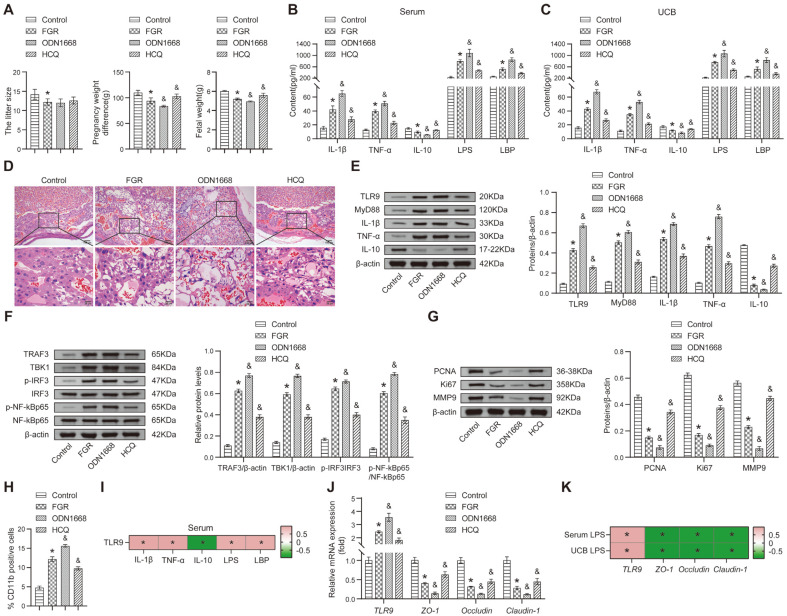
TLR9 could exacerbate inflammation by mediating the expression of inflammatory cytokines. (**A**) The litter size, pregnancy weight and fetal weight were assessed. (**B**) ELISA was performed to detect the contents of IL-1β, TNF-α, IL-10, LPS and LBP in maternal rats' serum; (**C**) The level of relative inflammatory markers was measured using ELISA in cord blood; (**D**) HE staining was employed to assess morphological changes in placental tissue; (**E**) The abundance of relative proteins via Western blot; (**F**) The effects of ODN1668 and HCQ on TRAF3, TBK1, IRF3, and NF-κBp65 expression in placental tissue were determined by Western blot; (**G**) The abundance of PCNA, Ki67, and MMP9 via Western blot; (**H**) The effects of ODN1668 and HCQ on neutrophils number were assessed by flow cytometry; (**I**) The correlation between the expression of TLR9 and relative cytokines were analyzed by Pearson's correlation; (**J**) Relative mRNA levels of *TLR9*, *ZO-1*, *Occludin*, and *Claudin-1* were detected by qRT-PCR; (**K**) Heatmap showing the correlation of TLR9, ZO-1, Occludin and Claudin-1 with LPS. *p < 0.05 compared with the Control group. &*p* < 0.05 compared with the FGR group. In the heatmaps, *p < 0.05 indicates a significant correlation between variables.

To explore the potential molecular mechanism by which TLR9 influences inflammation in FGR rats, we established additional animal groups with ODN1668 and HCQ pretreatment. FGR rats showed decreased pregnancy weight difference, litter size, and fetal weight relative to the Control group (Fig. 3A). ODN1668 decreased the pregnancy weight difference and fetal weight in FGR rats but did not significantly impact the litter size. In contrast, HCQ increased the pregnancy weight difference and fetal weight in FGR rats without significantly affecting the litter size (Fig. 3A). Furthermore, ODN1668 increased the levels of premature IL-1β, TNF-α, LPS and LPB relative to the FGR group but lowered IL-10 levels in FGR rats (Figs. 3B, 3C, and 3E). Compared to the FGR group, HCQ downregulated premature IL-1β, TNF-α, LPS, and LPB levels and upregulated IL-10 levels (Figs. 3B, 3C, and 3E). Additionally, we observed abundant necrosis cells, inflammatory cell infiltration and erythrocyte extravasation in the placental tissue of FGR rats which was exacerbated by ODN1668, while HCQ pretreatment partially mitigated these pathological phenomena (Fig. 3D).

In addition, our results showed that ODN1668 increased TLR9 and MyD88 expression while HCQ significantly decreased their expression levels in FGR rats compared to the FGR group (Fig. 3E). In addition, compared with the FGR group, ODN1668 increased the levels of TRAF3 and TBK1 and the ratio of p-IRF3/IRF3 and p-NF-kBp65/NF-kBp65. Comparatively, HCQ decreased the levels of TRAF3 and TBK1 and the ratio of p-IRF3/IRF3 and p-NF-kBp65/NF-kBp65 in FGR rats (Fig. 3F). We also observed that the expression of PCNA, Ki67 and MMP9 was decreased in the ODN1668 group but was increased in the HCQ group, compared to the FGR group (Fig. 3G). The proportion of CD11b-positive cells was increased in FGR rats compared to the Control group, and ODN1668 further increased the proportion, while HCQ inhibited the proportion of CD11b positive cells in FGR rats (Figs. 3H and S1B). Further, we analyzed the correlation between TLR9 expression and serum inflammation indicators using Pearson's correlation, and the results showed that with the increased expression of TLR9, the levels of LPS, LPB, premature IL-1β and TNF-α gradually increased, indicating that they are positively correlated. TLR9 was found to be negatively correlated with IL-10 (Fig. 3I). Thus, these findings indicated that TLR9 could aggravate inflammation by mediating the expression of inflammatory cytokines and affecting the development of FGR.

The above experimental studies indicated an increase in the level of LPS in FGR rats, which could be regulated by ODN1668 and HCQ. Considering that chronic inflammatory response induced by endogenous LPS may cause intestinal microbial dysbiosis and changes in intestinal permeability[38], we sought to investigate the effects of TLR9 on intestinal permeability in FGR rats. We found that *TLR9* levels were increased in the jejunum of FGR rats compared to the Control group, while the levels of *ZO-1*, *Occludin* and *Claudin-1* were decreased (Fig. 3J). In addition, ODN1668 upregulated *TLR9* and downregulated *ZO-1*, *Occludin* and *Claudin-1* compared to the FGR group, while the opposite was observed with HCQ, which reversed the levels of these factors in FGR rats (Fig. 3J). The correlation of these factors with LPS levels in the serum and cord blood was also analyzed, and the results indicated that LPS was positively correlated with TLR9 and negatively correlated with ZO-1, Occludin and Claudin-1 (Fig. 3K). Taken together, our findings suggest that TLR9 regulates the permeability of the intestinal barrier in FGR rats and that an increase in TLR9 in the jejunum may adversely affect the intestinal barrier function.

### HCQ Affects Gut Microbiota Diversity in FGR Female Rats

**Fig. 4:**
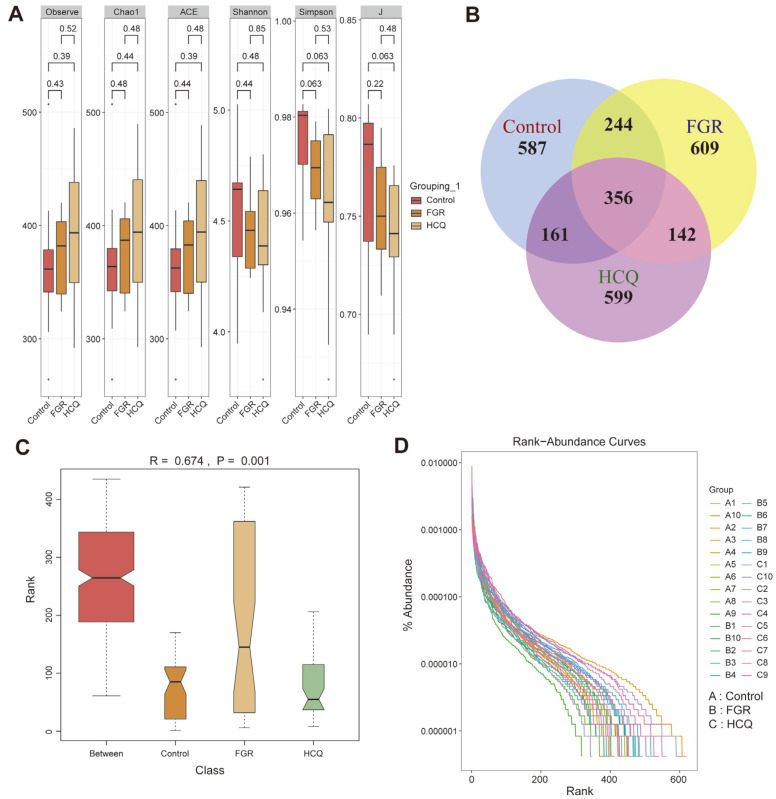
Effects of HCQ on gut microbiota diversity in FGR rats. (**A**) The box plot shows the trend of the Alpha diversity indexes; (**B**) The Venn diagrams show the clustering of operational taxonomic units in samples; (**C**) ANOSIM analysis was performed to assess the differences among samples. The vertical axis represents the rank between samples. The abscissa indicates the results between groups, and others indicate the results within each group; (**D**) Rank-abundance curve illustrating the species diversity of samples. The rank abundance curves were determined by ranking the ASVs in order of relative abundance from largest to smallest, with the degree of ASV as the horizontal coordinate and the relative percentage of the number of sequences contained in each ASV as the vertical coordinate.

The aforementioned experimental studies have demonstrated that HCQ can potentially alleviate inflammatory responses in female FGR rats, and we speculated that these physiological changes might be closely associated with alterations in the gut microbiota composition. To investigate this further, we collected feces samples and analyzed the changes in the gut microbiota composition in the FGR models using 16S rRNA sequencing. The analysis of the alpha diversity index revealed no significant differences among the three groups for all six indices (Fig. 4A). However, upon examining the Venn plot, we discovered that the Control group had 587 individual ASVs, while the FGR and HCQ groups had 609 and 599 individual ASVs, respectively. Interestingly, 356 ASVs were identical across all three groups (Fig. 4B). Furthermore, we analyzed the shared or unique microbiota at the genus level and above (Fig. S1C) and found no discernible differences in the microbiota specific to the Control and FGR groups at the phylum and class levels. Additionally, at the order level, there were 43, 43, and 42 ASVs in the Control, FGR and HCQ groups, respectively, with a total of 38 ASVs. When examining the family level, the Control, FGR and HCQ groups exhibited 68, 70, and 64 ASVs, respectively, with a total of 59 ASVs. Lastly, at the genus level, the Control, FGR and HCQ groups displayed 133, 133, and 132 ASVs, respectively, with a total of 113 ASVs. These results suggest significant differences in the gut microbiota composition among the three groups, specifically at levels below the order and above the genus.

An ANOSIM analysis was conducted to analyze the differences among samples. The results showed that the differences between groups (*r* =0.674, *P* = 0.0001) were greater than those within groups, indicating significant grouping (Fig. 4C). The Rank-abundance curve revealed that the Control group had the widest horizontal axis range, while the curve for the HCQ group was closer to that of the Control group, suggesting similarity in species (Fig. 4D). Hence, we could determine that the occurrence of FGR was linked to gut microbiota disturbance, and HCQ treatment may have a therapeutic effect by altering the structure of specific gut microbiota.

### HCQ Affects the Abundance of Specific Microbiota in FGR Female Rrats

**Fig. 5:**
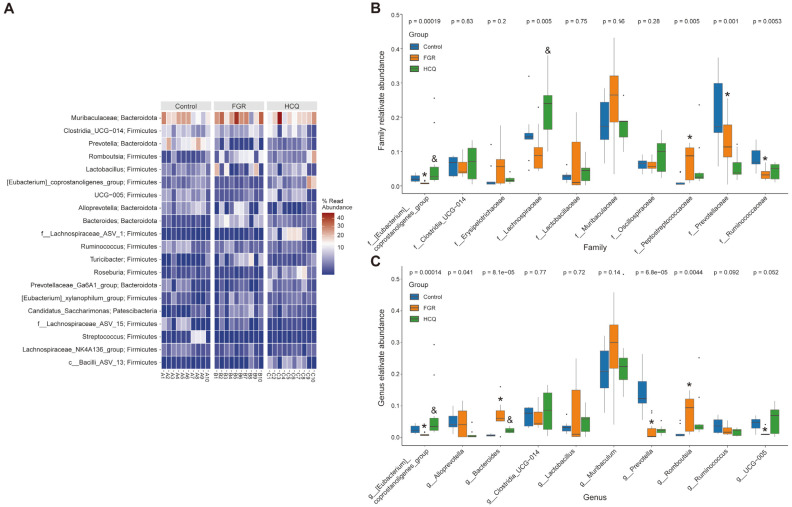
The abundance of gut microbiota was analyzed. (**A**) Heat map showng the microbial species distribution; (**B**) Abundance differences of bacterial categories were analyzed at the family level; (**C**) Relative abundance of microbiota at the genus level. *p < 0.05 compared with the Control group. & *p* < 0.05 compared with the FGR group.

We examined the abundance of the top 20 microbial taxa and analyzed the differences in the abundance of specific microbial taxa across samples. The heat map showed significant enrichment of *Muribaculaceae* in each sample, with higher abundance in the FGR group compared to the Control group. However, this enrichment could be reduced by HCQ pretreatment (Fig. 5A). At the family level, we further analyzed the abundance differences in specific microbiota and found that the abundance of *Eubacterium_coprostanoligenes_group* (*P* = 0.00019), *Lachnospiraceae* (*P* = 0.005), *Peptostreptococcaceae* (*P* = 0.005), *Prevotellaceae* (*P* = 0.001), and *Ruminococcaceae* (*P* = 0.0053) showed significant group differences. Further, compared with the Control group, the abundance of *Ruminococcaceae* (*P* = 0.0019), *Prevotellaceae* (*P* = 0.041) and *Eubacterium_coprostanoligenes_group* (*P* = 0.0012) displayed a downward trend in the FGR group, while that of *Peptostreptococcaceae* (*P* = 0.0032) increased. Although the abundance of these microbiotas could be reversed after HCQ pretreatment, only *Eubacterium_coprostanoligenes_group* (*P* = 0.00029) exhibited significant differences (Fig. 5B). At the genus level, we observed group differences in the abundance of *Eubacterium_coprostanoligenes_group* (*P* = 0.00014), *Alloprevotella* (*P* = 0.041), *Bacteroides* (*P* = 8.1e-05), *Prevotella* (*P* = 6.8e-05), and *Romboutsia* (*P* = 0.0044). The abundance of *Prevotella* (*P* = 0.00038), UCG.005 (*P* = 0.0065), and *Eubacterium_coprostanoligenes_group* (*P* = 0.0012) decreased in the FGR group, while that of *Romboutsia* (*P* = 0.0025) and *Bacteroides* (*P* = 0.0025) increased. In addition, although the abundance of these microbiota could be reversed after HCQ pretreatment, only *Eubacterium_coprostanoligenes_group* (*P* = 0.00021) and *Bacteroides* (*P* = 0.0025) exhibited significant differences (Fig. 5C). These findings suggest that HCQ could regulate the abundance of specific gut microbiota in FGR rats by restoring the aforementioned taxa to levels similar to those observed in the Control group.

### Correlation Analysis between the Expression of Relative Cytokines and Gut Microbiota

**Fig. 6:**
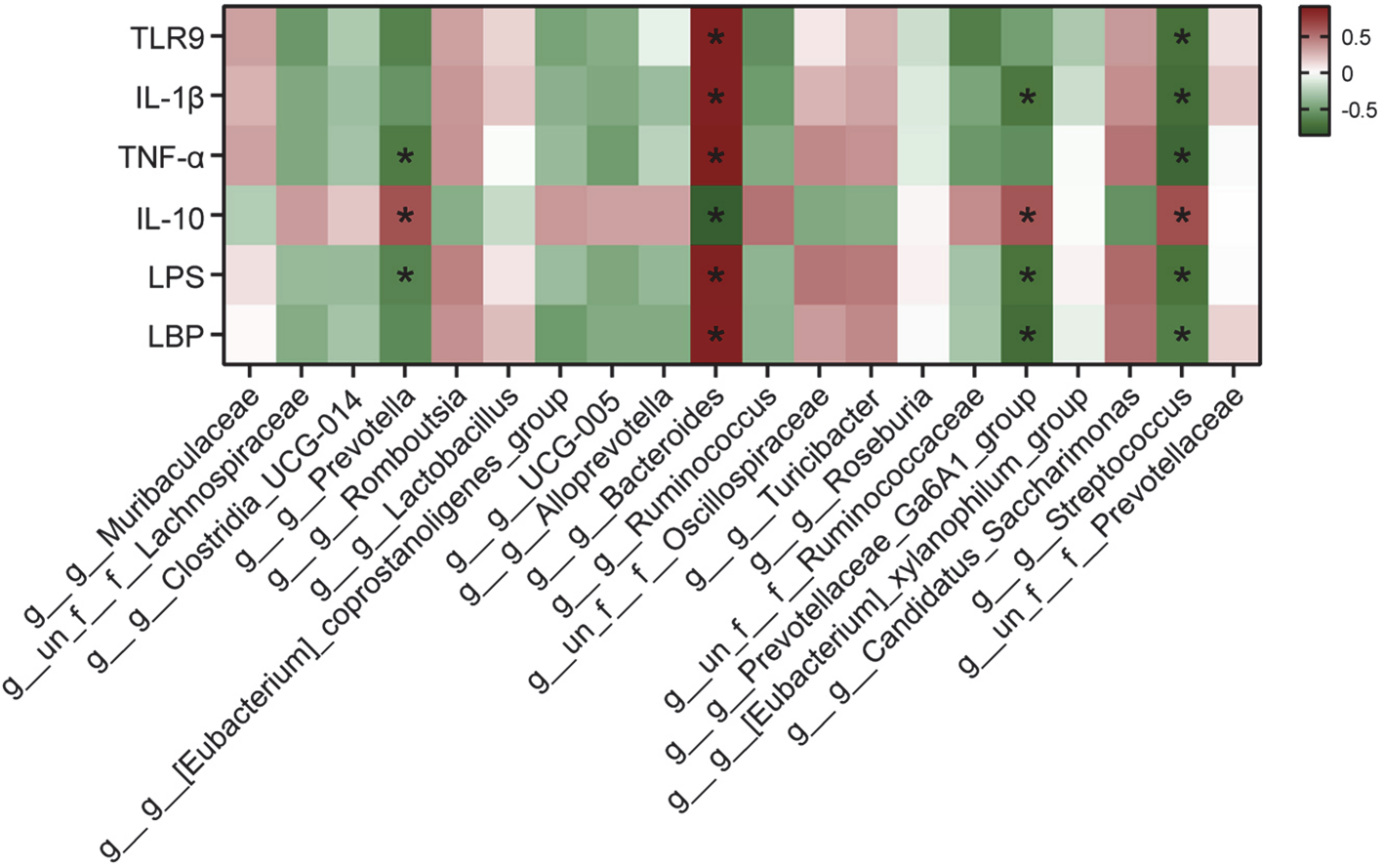
Heat map showing the correlation between the abundances of relative factors and the top 20 gut microbiota. Each small square represents a correlation. The color from red to green implies the intensity of the correlation from small to large. *p < 0.05 indicated a significant correlation between variables.

We analyzed the correlation between dominant gut microbiota at the genus level and the expression of TLR9-mediated cytokines in the serum from experimental rats. As shown in Fig. 6, TLR9 demonstrated a significant positive correlation with *Bacteroides* (*r* = 0.862, *P* = 0.005) and a significant negative correlation with *Streptococcus* (*r* = -0.695, *P* = 0.045). Premature IL-1β showed a positive correlation with *Bacteroides* (*r* =0.860, *P* = 0.001) and negative correlation with *Streptococcus* (*r* = -0.720, *P* = 0.011) and *Prevotellaceae_Ga6A1_group* (*r* = -0.664, *P* = 0.022). Further, TNF-α exhibited a significant positive correlation with *Bacteroides* (*r* =0.888, *P* = 0.0002) and negative correlations with *Prevotella* (*r* = -0.643, *P* = 0.028) and *Streptococcus* (*r* = -0.755, *P* = 0.006). The anti-inflammatory factor IL-10 displayed a positive correlation with multiple bacterial categories, including *Prevotella* (*r* = 0.615, *P* = 0.037), *Prevotellaceae_Ga6A1_group* (*r* = 0.601, *P* = 0.043), and *Streptococcus* (*r* = 0.629, *P* = 0.032), while negative correlations were observed with *Bacteroides* (*r* = -0.825, *P* = 0.002). In addition, LPS was found to be associated with four taxa, namely *Prevotella* (*r* = -0.601, *P* = 0.043), *Bacteroides* (*r* = 0.867, *P* = 0.017), *Prevotellaceae_Ga6A1_group* (*r* = -0.685, *P* = 0.043) and *Streptococcus* (*r* = -0.685, *P* = 0.017). Additionally, the correlation between LBP and *Bacteroides* (*r* = 0.860, *P* = 0.001), *Prevotellaceae_Ga6A1_group* (*r* = -0.720, *P* = 0.011) and *Streptococcus* (*r* = -0.629, *P* = 0.032) was also found to be significant. Taken together, our results suggest that TLR9 could regulate the level of inflammatory cytokines, LPS and LBP by modulating specific gut microbiota, thereby influencing the development of FGR.

### Fecal Microbiota from FGR Rats Disrupts the Protective Effect of HCQ on FGR Rats

**Fig. 7:**
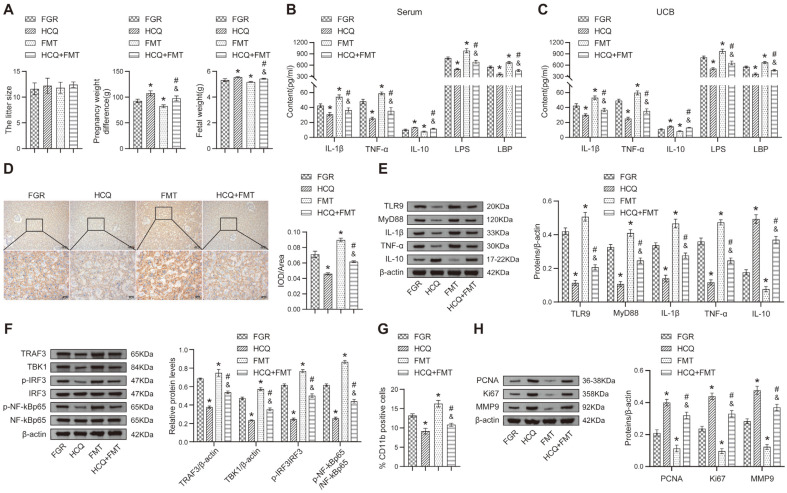
TLR9 regulates the pathogenesis of FGR through the gut microbiota. (**A**) The litter size, pregnancy weight, and fetal weight were determined. (**B**) ELISA was performed to examine the contents of IL-1β, TNF-α, IL-10, LPS and LBP in maternal rats' serum; (**C**) ELISA was applied to measure relative inflammatory marker levels in cord blood; (**D**) IHC staining was performed to assess the expression of TLR9 in placental tissue; (**E**) The abundance of relative proteins was shown by Western blot; (**F**) The effects of ODN1668 and HCQ on TRAF3, TBK1, IRF3, and NF-κBp65 expression in placental tissue were determined by Western blot; (**G**) The number of CD11b-positive cells was assessed by flow cytometry; (**H**) The abundance of PCNA, Ki67, and MMP9 was assessed by Western blot. *p < 0.05 compared with the FGR group. &*p* < 0.05 compared with the HCQ group. # *p* < 0.05 compared with the FMT group.

Here, we administered fecal microbiota from FGR rats to rats in the HCQ group through FMT and assessed the development of FGR. The FMT group exhibited reduced pregnancy weight difference and fetal weight compared to the FGR group, and the HCQ+FMT group displayed further decreases in pregnancy weight difference and fetal weight compared to the HCQ group. Additionally, the HCQ group showed increased pregnancy weight difference and fetal weight compared to the FMT group (Fig. 7A). However, there were no significant differences in litter size between the groups. Furthermore, FMT from FGR rats led to elevated levels of premature IL-1β, TNF-α, LPS and LBP and reduced IL-10 levels in the maternal serum and cord blood of rats in the HCQ group. HCQ treatment resulted in decreased premature IL-1β, TNF-α, LPS, and LBP levels, as well as increased IL-10 levels in the FMT group compared to the FMT group (Figs. 7B and 7C). IHC staining showed that TLR9 expression was increased in the placental tissues of rats in the HCQ group following FMT, whereas HCQ decreased TLR9 expression in the FMT group (Fig. 7D). The HCQ+FMT group exhibited higher protein levels of TLR9, MyD88, premature IL-1β and TNF-α compared to the HCQ group, while IL-10 was lower. HCQ treatment reduced the protein levels of TLR9, MyD88, premature IL-1β and TNF-α and increased IL-10 abundance compared to the FMT group (Fig. 7E). Additionally, the HCQ+FMT group showed increased expression of TRAF3 and TBK1 and elevated ratios of p-IRF3/IRF3 and p-NF-κB compared with the HCQ group. HCQ decreased TRAF3 and TBK1 expression and the ratio of p-IRF3/IRF3 and p-NF-κB compared to the FMT group (Fig. 7F). Furthermore, FMT increased the proportion of CD11b-positive cells compared with the HCQ group, while HCQ reduced CD11b-positive cells compared to the FMT group (Figs. 7G and S1D). In the HCQ+FMT group, PCNA, Ki67 and MMP9 expression was reduced compared to the HCQ group, whereas their levels were increased compared to the FMT group (Fig. 7H). Collectively, our findings suggest that transferring gut microbiota from FGR rats exacerbates the inflammatory response in FGR rats and counteracts the protective effect of HCQ in FGR rats and that HCQ attenuates FGR progression in FGR rats after FMT intervention.

## Discussion

The smooth progression of pregnancy is dependent on several factors, including the ability to defend against microorganisms. Maternal infection complicated with microbes may increase the risk of adverse outcomes such as preterm birth, miscarriage, and fetal growth restriction [39]. Therefore, it is of great significance to explore the pathogenesis of pregnancy complications. This study aimed to analyze the levels of inflammatory cytokines and the structure of gut microbiota in the FGR model, while also examining the potential role of TLR9 in mitigating abnormal inflammation and gut microbiota disturbances.

Maternal infection during pregnancy can trigger a systemic inflammatory response, resulting in changes in inflammatory cytokines and chemokines [40]. In this present study, we observed that FGR rats exhibited lower pregnancy weight, litter size, and fetal weight. Furthermore, we noted increased expression of pro-inflammatory cytokines and decreased levels of anti-inflammatory cytokines, indicating the presence of abnormal inflammation in FGR rats. Elevated levels of endogenous LPS and LBP were also observed in FGR rats. Studies have shown that the TLR family members play critical roles in the regulatory networks of inflammatory diseases [41, 42]. They can transmit signals through both MyD88-dependent and MyD88-independent pathways upon pathogen detection. Activation of the MyD88-dependent pathway leads to the activation of the transcription factor nuclear factor kappa B (NF-κB), resulting in the production of pro-inflammatory cytokines. During pregnancy, the innate immune system plays a crucial role in protecting the mother from various aggressions. Notably, neutrophil numbers steadily increase in the peripheral blood of pregnant women, serving as the first line of defense against bacterial and pathogenic attacks [43]. During pregnancy complications, there is an amplification of neutrophil numbers in the systemic circulation and decidua [44]. In this research, we observed an elevation in the number of neutrophils in the peripheral blood of FGR rats, which could contribute to inflammation. Additionally, TLRs play a crucial role in promoting antiviral innate immunity by stimulating the production of interferons and pro-inflammatory cytokines through the TRIF-TRAF3-TBK1-IRF3 pathway [45, 46]. Our results showed that the expression of TRAF3 and TBK1, as well as the phosphorylation of IRF3, were enhanced in FGR rats. During pregnancy, TLR2 and TLR4 are known to have prominent roles due to their upregulation in placental villi and trophoblasts [47]. The activation of TLR4/MyD88 signaling by LPS, in cooperation with LBP, triggers inflammatory responses [48]. However, in our experimental results, we found that the levels of TLR2 and TLR4 in the placental tissue were similar between FGR rats and healthy rats, while the content of TLR9 was increased, suggesting that TLR9 may play a more significant role in the pathogenesis of FGR. The preferential response of TLR9 in FGR may involve different signaling cascades, and the presence of endogenous LPS can induce chronic inflammatory responses, leading to dysbiosis in the gut microbiota and changes in intestinal permeability[38]. These changes allow LPS to enter the systemic circulation and interact with TLRs, leading to the eventual development of central and peripheral cytokine storms [49]. TLR9 is primarily located in endosomes, where it interacts with mtDNA and relies on MyD88 for immune responses [50]. The interaction between mtDNA and TLR9 stimulates NF-κB signaling and transcription of pro-inflammatory cytokines [51]. Previous studies have reported that the pathogenesis of FGR is associated with mitochondrial dysfunction [52]. Therefore, we speculate that in FGR, TLR9 may exhibit a more rapid response to other stimuli in addition to endogenous LPS signaling. However, further experimental evidence is required to confirm this speculation.

To gain a deeper insight into the involvement of TLR9 in the pathogenesis of FGR, we performed experiments using a human trophoblast cell line called HTR-8/Svneo with TLR9 activator and inhibitor, which plays a crucial role in pregnancy [53]. Our findings revealed that TLR9 overexpression resulted in reduced proliferation and invasion abilities of HTR-8/Svneo cells compared to the Control group, while knockdown of TLR9 showed an opposite trend, thereby suggesting that TLR9 silencing can promote trophoblast cell proliferation and invasion, consistent with the results in a previous study [54]. In addition, we administered ODN1668 (a TLR9 activator) and HCQ (a TLR9 inhibitor) to FGR rats. HCQ administration led to increase in the pregnancy weight difference and fetal weight compared to the FGR group, while ODN1668 resulted in decreased pregnancy weight difference and fetal weight. The HCQ group exhibited significantly reduced levels of pro-inflammatory cytokines and inflammation compared to the FGR group, consistent with the in vitro results. On the other hand, ODN1668 administration elevated the levels of pro-inflammatory cytokines and inflammation in FGR rats. Furthermore, TLR9 levels were positively correlated with pro-inflammatory cytokines and negatively correlated with IL-10 levels.

In the FGR model, the administration of ODN1668 activated the TRAF3-TBK1-IRF3 pathway, while HCQ inhibited this signaling pathway. Furthermore, HCQ treatment increased the expression of PCNA and Ki67, which are involved in proliferation, as well as MMP9, which is involved in cell migration in FGR rats. These findings suggest that TLR9 could promote the development of FGR by stimulating MyD88 to regulate the levels of inflammatory factors. Additionally, our experiments showed that HCQ inhibited the elevated levels of LPS and LBP in the serum and cord blood of FGR rats, which might be attributed to the reduced TLR9-mediated neutrophil responses, thereby inhibiting neutrophil activation triggered by LPS [55, 56]. Our results also demonstrated that ODN1668 increased neutrophil numbers, whereas HCQ reduced them in FGR, indicating that TLR9 regulates neutrophil activation. Neutrophils can enhance their antimicrobial properties by releasing neutrophil extracellular traps (NETs) [57], which can also contribute to pathological damage through inflammatory responses [58]. However, further investigation is required to understand the potential regulatory network of TLR9-mediated neutrophil activation.

Numerous studies conducted in recent decades have consistently demonstrated a connection between altered microbiota structure and the development of various pregnancy complications [59, 60]. Members of the TLR family play a crucial role as sensors of the gut microbiota, maintaining gut homeostasis and regulating the balance between the host immune system and gut microbiota [61]. Bacteria and inflammation can alter intestinal permeability [62]. Therefore, we hypothesized that the promoting effect of TLR9 on the development of FGR is closely related to the gut microbiota. Our findings indicated that TLR9 levels were increased in the jejunum of FGR rats and positively correlated with LPS. Additionally, we observed a decrease in the expression of tight junction proteins in the jejunum of FGR rats, leading to disruption of the intestinal barrier. The expression of these proteins was negatively correlated with LPS. ODN1668 treatment inhibited the expression of tight junction proteins, while HCQ treatment elevated their levels in FGR rats. These results suggest that silencing TLR9 suppresses the increase in intestinal permeability observed in FGR rats.

Alterations in gut microbial composition are known to contribute to intestinal barrier dysfunction [63]. Probiotic treatment has shown the potential to reduce gut dysbiosis, improve intestinal leakage, and reduce inflammatory activation [64]. In our study, the analysis of gut microbiota in female rats using 16S rRNA sequencing revealed that FGR rats exhibited gut microbiota disturbances compared to healthy rats. Specifically, we observed an increase in the abundance of *Bacteroides* in the FGR group, consistent with findings from a previous study [65]. The abundance of probiotics (*Prevotella*), UCG.005 and *Eubacterium_coprostanoligenes_group* was decreased in the FGR group, while that of *Romboutsia* was increased. However, HCQ treatment could restore the levels of these genera to levels similar to those in the Control group, indicating that TLR9 could be a new target for improving the gut microbiota structure in FGR rats. Previous studies have reported that TLR9 regulates inflammation and bone loss by affecting the structure of the gut microbiota [66]. Thus, we analyzed the correlation between the top 20 gut microbiota taxa at the genus level and inflammatory factors and TLR9. The results indicated that *Bacteroides* displayed a significant positive correlation with the abundance of TLR9, pro-inflammatory factors IL-1β and TNF-α, as well as LPS and LBP, and a negative correlation with IL-10. *Streptococcus* was negatively associated with TLR9 and pro-inflammatory cytokines and positively associated with IL-10. Previous studies have reported that changes in the abundance of *Bacteroides* and *Streptococcus* affect the development of TLR9-mediated colitis and influenza [67, 68]. These results suggest that *Bacteroides* and *Streptococcus* may have a greater influence on TLR9-mediated inflammatory and immune responses. Additionally, we showed that *Prevotella* and *Prevotellaceae_Ga6A1_group* were correlated with IL-1β, TNF-α and IL-10, indicating that these taxa could also affect TLR9-mediated inflammatory response in FGR rats. The immune regulatory role of IL-10 in maintaining intestinal homeostasis has been well established [69]. Our findings suggest that IL-10 could be closely associated with various bacterial taxa in the gut of rats, indicating a possible involvement of IL-10 in immune regulation in FGR rats. Thus, further investigations are required to provide more clarification on these findings.

FMT has emerged as a promising therapeutic approach for modulating the gut microbiota in recipient. This strategy has shown success in treating various disorders, including gastrointestinal disorders [70], neurological disorders [71], and cancers [72]. Previous studies have demonstrated that FMT can rescue perfluorooctanoic acid-induced FGR by counteracting the gut microbial dysbiosis in FGR animals [73]. In our study, we observed that the gut microbiota from FGR rats disrupted the therapeutic effect of HCQ in FGR rats. Specifically, the transfer of gut microbiota from FGR rats resulted in a reduction in the pregnancy weight difference and fetal weight, an increase in levels of pro-inflammatory factors, and a decrease in the expression of anti-inflammatory factors and pro-cell growth factors. Moreover, the gut microbiota from FGR rats activated the TRAF3-TBK1-IRF3 pathway and induced neutrophil activation in FGR rats. Furthermore, FMT intervention after HCQ treatment led to an improvement in the pathological symptoms of FGR rats. These results provide evidence supporting that TLR9 silencing can regulate the gut microbiota to ameliorate FGR.

## Conclusion

In summary, our study presents a potential therapeutic approach for potentially improving the management of FGR. We showed that TLR9 could effectively mitigate the abnormal inflammatory response in FGR rats by modulating the levels of inflammatory cytokines. Additionally, we observed that the downregulation of TLR9 was associated with the improvement of gut microbiota disturbance in FGR rats. The results of FMT intervention also highlight the crucial role of gut microbiota in interfering with the therapeutic efficacy of HCQ. Therefore, the TLR9-mediated regulation of inflammation and gut microbiota in FGR provides valuable insights into the development of novel strategies for the prevention and treatment of FGR.

## Supplemental Materials

Supplementary data for this paper are available on-line only at http://jmb.or.kr.

## Figures and Tables

**Table 1:** Primer sequences.

Gene	F (5'-3')	R (5'-3')
*TLR2*	GGAGGTCTCCAGGTCAAATCT	AGTCACCATGGCCAATGTAGG
*TLR4*	GGCTTCTAACCTCAACGACCT	ATGATTCTTTGCCTGAGTTGCTT
*TLR9*	CCTGGCTAACGGTGTGAAGT	GGGTCAGCAAAGGTAGCCAT
*MyD88*	ACTGTATGAACTGAAGGACCGCATC	ACTCCTGTTTCTGCTGGTTGCGTA
*IL-1β*	CAGCAGCATCTCGACAAGAG	AAAGAAGGTGCTTGGGTCCT
*TNF-α*	CCCCTCTATTTATAATTGCACCT	CTGGTAGTTTAGCTCCGTTT
*IL-10*	AATAAGCTCCAAGACAAAGGT	TCACGTAGGCTTCTATGCAG
*ZO-1*	CTTGCCACACTGTGACCCTA	ACAGTTGGCTCCAACAAGGT
*Occludin*	TTCTGTGCTCACAGGTGGTT	TGGGCTGGATGCCAATTTAGT
*Claudin-1*	GCCCCAATGGAAGATTTACTCCT	CTGTATCTGCCCGGTGCTTT
*β-actin*	ACATCCGTAAAGACCTCTATGCC	TACTCCTGCTTGCTGATCCAC
