# Supplementary material for: The Gut Microbiota of Pregnant Rats Alleviates Fetal Growth Restriction by Inhibiting the TLR9/MyD88 Pathway
Source: J Microbiol Biotechnol. 2023 Jun 30;33(9):1213–27. doi: 10.4014/jmb.2304.04020 (PMC10580896; doi:10.4014/jmb.2304.04020)
Supplement: Supplementary file 1 [file jmb-33-9-1213-supple.pdf]

J. Microbiol. Biotechnol.
https://doi.org/10.4014/jmb.2304.04020

Supplementary Figure

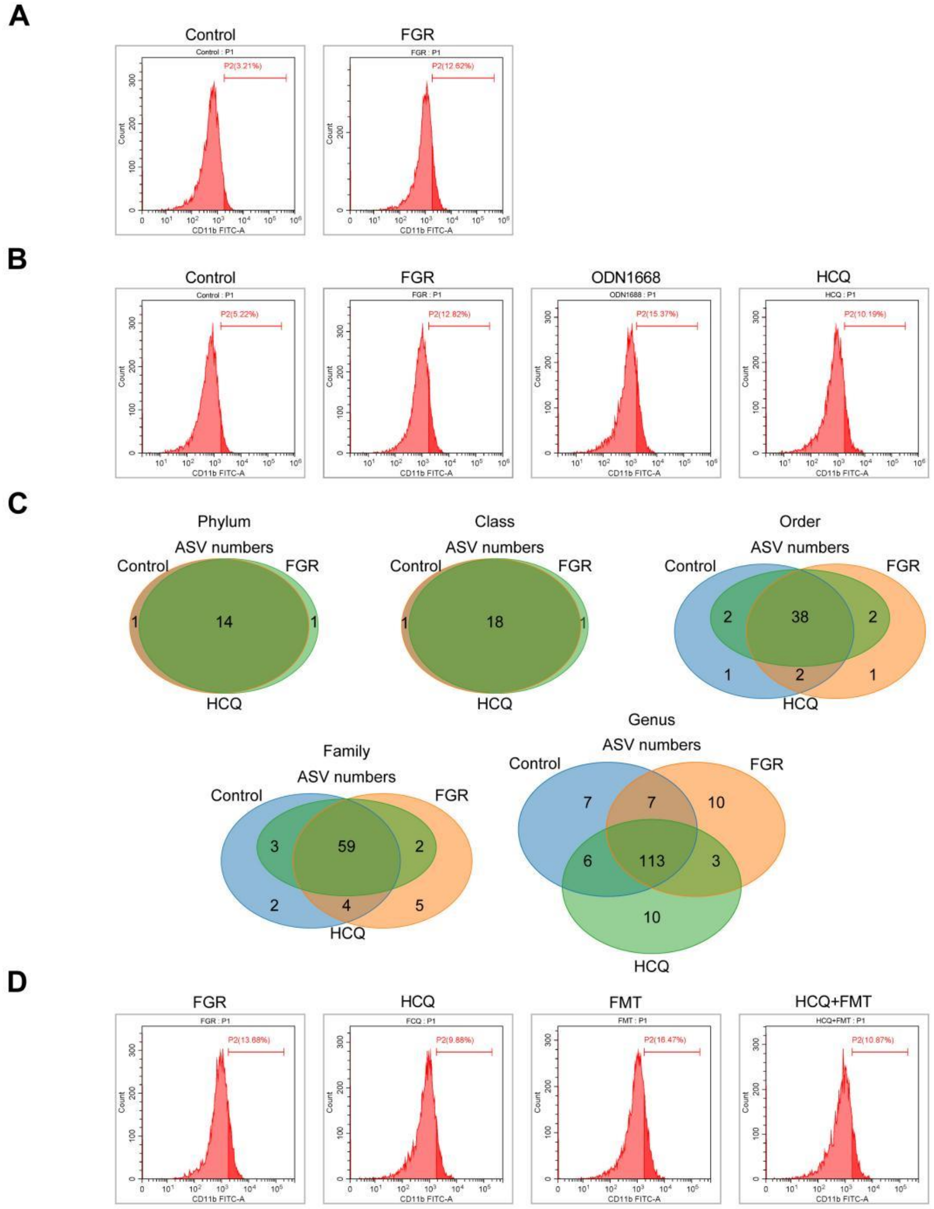

Supplement Figure 1. Results of flow cytometry and classification above the genus level. (A) Representative flow cytometry images of the Control and FGR groups; (B) Representative flow cytometry images of the Control, FGR, ODN1668, and HCQ groups; (C) Venn diagram showing classification of microbiota above the genus level; (D) Representative flow cytometry images of the FGR, HCQ, FMT, and HCQ+FMT groups.
